# An open-label, randomized, phase II trial evaluating the efficacy and safety of standard of care with or without bevacizumab in platinum-resistant epithelial ovarian, fallopian tube, or primary peritoneal cancer patients previously treated with bevacizumab for front-line or platinum-sensitive ovarian cancer: rationale, design, and methods of the Japanese Gynecologic Oncology Group study JGOG3023

**DOI:** 10.1186/s12885-018-4505-4

**Published:** 2018-07-31

**Authors:** Tadahiro Shoji, Shinichi Komiyama, Junzo Kigawa, Hiroshi Tanabe, Kazuyoshi Kato, Hiroaki Itamochi, Hiroyuki Fujiwara, Shoji Kamiura, Tetsutaro Hamano, Toru Sugiyama, for the Japanese Gynecologic Oncology Group

**Affiliations:** 1 Department of Obstetrics and Gynecology, Iwate Medical University School of Medicine, 19-1 Uchimaru, Morioka, Iwate 020-8505 Japan; 2 Department of Gynecology, Toho University Ohashi Medical Center, Tokyo, Japan; 3 Department of Obstetrics and Gynecology, Matsue City Hospital, Shimane, Japan; 4 Department of Obstetrics and Gynecology, The Jikei University School of Medicine, Kashiwa Hospital, Chiba, Japan; 5 Department of Gynecology, Cancer Institute Hospital, Tokyo, Japan; 6 Department of Obstetrics and Gynecology, Jichi Medical University, Tochigi, Japan; 7 Department of Gynecology, Osaka Medical Center for Cancer and Cardiovascular Diseases, Osaka, Japan; 8 Clinical Trial Coordinating Center, Kitasato Academic Research Organization, Kitasato University, Tokyo, Japan

**Keywords:** Bevacizumab, Platinum-resistant ovarian cancer, Ovarian cancer, Chemotherapy, Progression-free survival, Beyond progression, Continued treatment, Randomized clinical trial, Vascular endothelial growth factor

## Abstract

**Background:**

We present the study rationale and design of the JGOG3023 study, an open-label, parallel-arm, randomized, phase II trial that aimed to assess the efficacy and safety of chemotherapy with or without bevacizumab in patients with platinum-resistant recurrent epithelial ovarian, fallopian tube, or primary peritoneal cancer who were previously treated with bevacizumab for front-line or platinum-sensitive ovarian cancer. We hypothesize that patients treated with a combination of single-agent chemotherapy and bevacizumab will show improved progression-free survival (PFS) compared with those treated with single-agent chemotherapy alone, in the setting beyond disease progression following prior bevacizumab treatment.

**Methods/design:**

A total of 106 patients who have recurrence or progression of ovarian cancer, while receiving chemotherapy or within 6 months after the final dose of platinum, after completing at least three cycles of bevacizumab plus platinum chemotherapy will be randomized in a 1:1 ratio to treatment with single-agent chemotherapy or single-agent chemotherapy combined with bevacizumab. For chemotherapy, one of the following four drugs will be chosen by an investigator: pegylated liposomal doxorubicin, topotecan, paclitaxel, or gemcitabine. The primary endpoint is investigator-assessed PFS. The secondary endpoints are overall survival, objective response rate, number of paracentesis, and response rate by CA125. Safety will be evaluated by the incidence of adverse events.

**Discussion:**

This study will assess the efficacy and safety of bevacizumab in combination with single-agent chemotherapy, which could be used continuously after disease progression following standard platinum-based chemotherapy with bevacizumab.

**Trial registration:**

UMIN000017247 (registered April 22, 2015).

## Background

In Japan, ovarian cancer is diagnosed in nearly 9000 patients each year. The annual number of deaths due to ovarian cancer is more than 4500, and the incidence and mortality rates are increasing every year [1]. Few symptoms are noticed in the early stage of ovarian cancer. No appropriate diagnostic method is currently available; therefore, it is difficult to detect ovarian cancer at an early stage. More than half of ovarian cancer patients are diagnosed at advanced stages, such as stage III or further, which results in a poor prognosis. Treatment options include chemotherapy and surgery. Although surgery with or without adjuvant chemotherapy improves survival, most patients experience recurrence or relapse, and the outcome is still poor. In addition, although optimal debulking with initial surgery may be achieved in patients with advanced ovarian cancer, recurrence occurs in a high proportion of patients with optimal and suboptimal debulking. Because the survival time of ovarian cancer patients after recurrence is approximately 2 years [2] and cure is difficult to achieve following recurrence, improving quality of life and palliative care are also important treatment goals along with prolongation of survival time.

### Chemotherapy and molecular-targeted agents as standard of care for ovarian cancer

The combination of platinum and taxane has been established as the standard adjuvant chemotherapy for ovarian cancer [3]. Platinum-based combination therapy is also the main chemotherapy for advanced ovarian cancer. However, most patients who initially respond to platinum-based combination therapy present with recurrence or disease progression [4]. In patients resistant to platinum therapy who present with recurrence, non-platinum-containing chemotherapy, usually single-agent, is used to avoid toxicity; however, the duration of response to chemotherapy in recurrent ovarian cancer patients seldom exceeds that of the initial chemotherapy [5]. Treatment options for these platinum-resistant patients are limited, and are not particularly effective in prolonging survival. Currently, the molecular-targeted agent pazopanib [4] has shown improvement in progression-free survival (PFS) in various settings.

Bevacizumab, a recombinant humanized monoclonal antibody that limits angiogenesis by inhibiting vascular endothelial growth factor (VEGF) and the first molecular-target agent introduced in the gynecological field, has been established as a standard therapy for patients with ovarian cancer based on the prolongation of PFS shown in previous studies. For example, bevacizumab was given as first-line treatment [6] and to patients with platinum-sensitive recurrent epithelial ovarian, primary peritoneal, or fallopian tube cancer [7]. Furthermore, a combination treatment superior to single-agent chemotherapy in patients with platinum-resistant recurrent ovarian cancer was shown for the first time in the AURELIA study, an open-label, randomized, phase III trial [8]. The results showed an improved PFS (6.7 months vs. 3.4 months) and objective response rate (27.3% vs. 11.8%) in patients who received a combination of single-agent chemotherapy plus bevacizumab compared with standard single-agent chemotherapy.

### Study rationale

VEGF is one of the most predominant factors in tumor growth [9] and has an important role in the angiogenic pathway, which suggests that sustained VEGF inhibition by treatment through multiple lines (first- to second-line treatment) is essential for long-term disease control beyond progressive disease (PD) [10]. This hypothesis was confirmed in previous clinical trials on other tumor types, including colorectal cancer (the Treatment through Multiple Lines study [ML18147]) [11] and breast cancer (the TANIA study) [12]. Furthermore, it is currently being examined in three ongoing trials in patients with platinum-sensitive recurrent lung cancer [13], glioblastoma [14], and ovarian cancer (the MITO16MANGO2b study) [15], as opposed to the present trial, which includes platinum-resistant patients. Based on the results in breast and colorectal cancer patients, continued treatment with bevacizumab beyond PD is expected to improve therapeutic results of recurrent cancers, irrespective of the cancer type.

Previous studies in colorectal cancer patients showed a prolonged overall survival (OS) in patients who continuously received chemotherapy plus bevacizumab beyond PD from first- to second-line therapy compared with those who received chemotherapy alone [11, 16]. Similarly, prolonged PFS was shown in breast cancer patients who received chemotherapy plus bevacizumab treatment beyond PD compared with those who received chemotherapy alone [12]. The efficacy of bevacizumab treatment beyond PD, which has been verified in previous colorectal and breast cancer trials, supports the validity of the present study design.

Adverse events (AEs) reported with bevacizumab treatment include gastrointestinal perforation, delayed wound healing, tumor-related hemorrhage, pulmonary hemorrhage (hemoptysis), thromboembolism, hypertensive encephalopathy/hypertensive crisis, and posterior reversible encephalopathy syndrome [17]. In the ML18147 study, the most frequently reported grade 3 or more AEs among 409 colorectal cancer patients treated with chemotherapy plus bevacizumab were neutropenia (16%, 65/409), diarrhea (10%, 40/409), and asthenia (6%, 23/409), and 32% (129/409) of the patients presented serious AEs [11]. The TANIA study assessed the safety of continued treatment with bevacizumab in 245 breast cancer patients whose disease had progressed after treatment with bevacizumab plus chemotherapy, and the most common grade 3 or more AEs among the patients treated with chemotherapy plus continued treatment with bevacizumab were hypertension (13%, 33/245), neutropenia (12%, 29/245), and hand-foot syndrome (11%, 27/245), and 25% (61/245) of the patients presented serious AEs [12]. In the AURELIA study, the findings of grade ≥ 2 hypertension and proteinuria in patients who received chemotherapy combined with bevacizumab were consistent with those previously reported in patients with ovarian and other cancer types; however, this study was not designed to assess the safety of continued therapy with bevacizumab [8]. The safety of continued therapy with bevacizumab has been confirmed in colorectal cancer and breast cancer, but requires further evaluation in patients with ovarian cancer.

Among the AEs reported in ovarian cancer patients treated with bevacizumab, one of the most concerning is gastrointestinal perforation. The AVF2949g study [18], a single-arm phase II clinical study of bevacizumab monotherapy in patients with recurrent ovarian cancer (including both platinum-sensitive and platinum-resistant recurrent ovarian cancer), was discontinued because five of the 44 accumulated patients developed gastrointestinal perforation. Those five patients had received prior treatment with three regimens, and therefore, cautious use of bevacizumab is warranted in patients heavily pretreated with bevacizumab. Gastrointestinal infiltration that results from ovarian cancer metastasis was observed in 40 of 44 patients included in the study. Because many of the patients enrolled in the study originally had a potentially high risk of gastrointestinal perforation, the groups with or without development of gastrointestinal perforation were compared. However, no difference was observed between the groups other than the number of previous regimens. Rectovaginal nodularity, bowel surgery, and obstruction or ileus have been reported as risk factors for gastrointestinal perforation with bevacizumab in patients with recurrent ovarian cancer; however, it remains unclear whether the number of previous regimens constitutes a risk [19, 20]. Thus, risk factors of gastrointestinal perforation remain unclear. It is essential to identify the risks of gastrointestinal perforation by analyzing the results of a randomized study with a control group in order to determine the patient population in which bevacizumab can be used safely and effectively. It would also be useful to explore the correlation between the number of previous regimens and the incidences of gastrointestinal perforation.

Another reason for conducting the present study is that although the efficacy of bevacizumab in platinum-resistant recurrent ovarian cancer was previously shown in the AURELIA study, this study included few patients previously treated with bevacizumab. Therefore, it is unknown if the efficacy of bevacizumab reported in previous studies is also achieved in patients previously treated with bevacizumab.

Based on the above, the aim of this study is to assess the efficacy and safety of chemotherapy with or without bevacizumab in patients with platinum-resistant recurrent ovarian cancer who were previously treated with bevacizumab as a first-line therapy or patients who have platinum-sensitive recurrent ovarian cancer. We hypothesize that patients treated with a combination of single-agent chemotherapy and bevacizumab will have improved PFS compared with those treated with single-agent chemotherapy alone, in the setting beyond disease progression following prior bevacizumab treatment.

## Methods/design

### Study design and setting

The present study is designed as a multicenter, open-label, parallel-arm, randomized, phase II trial enrolling patients who have recurrence or progression of ovarian cancer while receiving chemotherapy or within 6 months after the final dose of platinum, after completing at least three cycles of bevacizumab plus platinum chemotherapy. If the present trial demonstrates statistical significance in the efficacy analysis as well as the safety to justify the planning of a phase III trial, a confirmatory phase III trial will be considered. In the present study, patients will be divided into two groups: single-agent chemotherapy (Group A) and single-agent chemotherapy combined with bevacizumab (Group B). The study is designed to determine whether the combined treatment is superior to the single-agent treatment in bevacizumab-pretreated platinum-resistant ovarian cancer patients.

Institutional review board approval has been obtained from each participating center; details of the relevant ethics committees are provided in the Supplementary Information. All patients will be required to provide signed informed consent prior to initiation of any study-specific procedures and treatment as confirmation of the patient’s awareness and willingness to comply with the study requirements. This trial will be conducted according to the International Conference on Harmonization Guidelines for Good Clinical Practice, the Ethical Guideline for Medical and Health Research involving Human Subjects and applicable local laws and regulations. The study was initiated on 1 June 2015.

### Study population

The inclusion criteria are as follows: patients aged 20 years or more with histologically confirmed epithelial ovarian, fallopian tube, or primary peritoneal carcinoma; with platinum-resistant disease, defined as progression within < 6 months from completion of a minimum of three platinum therapy cycles, including bevacizumab (assessment for disease progression by tumor marker alone is not accepted); with Eastern Cooperative Oncology Group Performance Status (ECOG PS) of 0–2; with Response Evaluation Criteria In Solid Tumors (RECIST) progression, with either measurable or nonmeasurable disease or those who can be evaluated based on Gynecological Cancer Intergroup (GCIG) CA125 criteria; with a life expectancy of ≥90 days; and with adequate organ function as determined by laboratory tests (neutrophil count ≥1500/mm^3^, platelet count ≥10.0 × 10^4^/mm^3^, hemoglobin ≥9.0 g/dL, total bilirubin < 1.2 mg/dL, aspartate aminotransferase/alanine aminotransferase < 100 IU/L and < 200 IU/L for those with liver metastasis, serum creatine ≤1.5 mg/dL, proteinuria ≤1+, and prothrombin time [international normalized ratio maximum] ≤1.5).

Patients will be excluded based on the following criteria: patients with ovarian borderline malignant tumor; with history of other clinically active malignancy within 5 years of enrollment; with ≥4 previous anticancer regimens; with a history of bowel obstruction, including subocclusive disease, related to the underlying disease and history of abdominal fistula, gastrointestinal perforation, or intra-abdominal abscess; with evidence of rectosigmoid involvement by pelvic examination or bowel involvement on computed tomography or clinical symptoms of bowel obstruction; with surgery within 28 days prior to the study start or anticipation of the need for major surgery during study treatment; with current or recent (within 10 days prior to the first study treatment dosing) chronic daily treatment with aspirin (≥324 mg/day) or clopidogrel (≥75 mg/day); with an anticipated need of palliative radiotherapy < 14 days prior to the study start; with left ventricular ejection fraction below 50% (only applicable for patients intended to be treated with pegylated liposomal doxorubicin); with pre-existing peripheral neuropathy greater than or equal to Common Toxicology Criteria [21] grade 2 who were planned to receive paclitaxel; with symptomatic central nervous system metastasis; with a history or evidence of thrombotic or hemorrhagic disorders, New York Heart Association grade II or greater congestive heart failure, serious cardiac arrhythmia requiring medication, uncontrolled hypertension, a nonhealing wound, ulcer, or bone fracture, serum positive for hepatitis B surface antigen (HBsAg[+]), hepatitis B core antibody, and/or HBsAb(+) and ≥ 2.1 log copies/mL of hepatitis B virus-DNA levels, or HIV(+); with current or recent treatment with another investigational drug within 30 days of the first study treatment dosing; with known hypersensitivity to any of the study drugs or excipients; pregnant or lactating women or those with childbearing potential not using highly-effective contraception; and patients judged inappropriate to participate in this study by the principal investigator.

Bevacizumab is the only targeted drug approved for treatment of ovarian cancer by the national health insurance coverage. Therefore, none of the patients will have previously used any targeted drug, including non-VEGF antiangiogenics. In Japan, about 20% of patients are treated with an initial dose-dense paclitaxel–carboplatin regimen. Therefore, a minority of subjects in this study will be enrolled for a recurrence within 6 months of receiving dose-dense paclitaxel–carboplatin.

### Study treatment and procedures

**Fig. 1:**
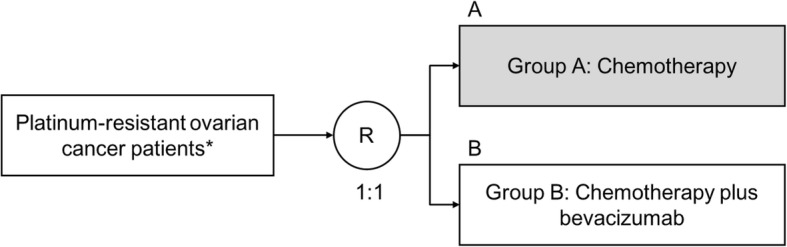
Study design. Bevacizumab-pretreated, platinum-resistant ovarian cancer patients will be randomized 1:1 to treatment with chemotherapy alone or chemotherapy plus bevacizumab. *Defined as progression within < 6 months from completion of a minimum of three platinum therapy (including bevacizumab) cycles

Group A will receive single-agent chemotherapy alone and Group B will receive single-agent chemotherapy combined with bevacizumab (Fig. 1). Before randomization, the chemotherapy agent will be chosen by an attending physician from the following four regimens: pegylated liposomal doxorubicin, topotecan, paclitaxel, or gemcitabine, which is indicated for platinum-resistant ovarian cancer in Japan [22]. The use of generic products for paclitaxel and gemcitabine will be allowed.

The drugs will be administered according to 1) package inserts of the drugs, 2) ovarian cancer treatment guidelines in Japan or elsewhere, and 3) clinical practices adopted in the actual clinical setting in each participating institution. The dosing schedule for each chemotherapy regimen will be as follows: pegylated liposomal doxorubicin will be administered intravenously (i.v.) at 40 mg/m^2^ or 50 mg/m^2^, 1 mg/min on Day 1 (with 28 days as one cycle); topotecan will be administered i.v. at 1.25 mg/m^2^ for more than 30 min on Days 1, 2, 3, 4, and 5 (with 21 days as one cycle); paclitaxel will be administered i.v. at 80 mg/m^2^ for 60 min on Days 1, 8, and 15 (with 21 days as one cycle); and gemcitabine will be administered i.v. at 1000 mg/m^2^ for 30 min on Days 1 and 8 (with 21 days as one cycle). Each cycle will be repeated until disease progression. Chemotherapy can be started at a reduced dose if it was already administered at a reduced dose in the previous treatment, and dose reduction criteria allowing two-phase reductions will be followed.

The treatment regimen of bevacizumab that will be given in combination with chemotherapy will be as follows: bevacizumab will be administered i.v. at 15 mg/kg on Day 1 (with 21 days as one cycle) for 90 min (at first administration), 60 min (at second administration if the first administration was well tolerated and more than 21 days passed since the first administration), 30 min (at third administration or thereafter if the second and previous administration was well tolerated and more than 21 days passed after the second and previous administration). If an interval between the first administration in this trial and the pre-trial previous administration is less than 21 days, the previous infusion time is to be followed. A cycle will be repeated until disease progression.

Discontinuation or modification of the chemotherapy regimens (i.e., drug dose change in response to toxicities or improving/worsening disease) will be based on the presence of AEs such as neutropenia, thrombocytopenia, hand-foot syndrome, and nonhematological toxicity, among others. The dose could also be reduced at the discretion of an attending physician, and once reduced, the dose will not be increased. If a dose reduction exceeding that defined in the protocol is necessary, the chemotherapy treatment will be discontinued.

The bevacizumab dose will not be reduced except for adjustments based on patients’ body weight. If bevacizumab is not administered for two consecutive cycles, the treatment with bevacizumab will be discontinued and patients will be treated with chemotherapy alone thereafter. If administration of chemotherapy is postponed because of AEs, administration of bevacizumab will continue unless bevacizumab must also be postponed.

Drugs other than those specified in the protocol of this trial will not be used. The following drugs will be prohibited: 1) anticancer treatment including other chemotherapy drugs than those defined in this trial, endocrine therapy, radiation therapy, hyperthermia therapy, surgery; 2) drugs not approved in Japan or those under clinical trials for new drug application; and 3) drugs and therapy thought to affect the safety and efficacy of the drugs of this trial. The following drugs will be allowed: 1) antiemetics, 2) treatment for AEs (including bisphosphonate and anti-RANKL antibody for the treatment of bone metastases), and 3) anticoagulants only for prophylactic use.

### Study endpoints

The primary efficacy endpoint will be investigator-assessed PFS, according to RECIST v1.1 or GCIG CA125 criteria. The response to chemotherapy will be assessed every 6 weeks. Because CT is not performed every two cycles, an adjustment to PFS is not necessary to control for cycle length for different single-agent chemotherapies. The secondary efficacy endpoints will be OS, objective response rate (ORR), number of paracentesis, and the response rate by tumor marker CA125. The secondary safety endpoint will be the incidence of AEs, as determined by the “Common Toxicity Criteria for Adverse Events v4.0 JCOG Japanese version” (CTCAE v4.0-JCOG) [21]. Additionally, because weekly paclitaxel has been reported to be effective in inhibiting angiogenesis [23, 24], it is possible that combination of paclitaxel with bevacizumab may strengthen the antitumor effect, which was first demonstrated in the AURELIA study [8]. Therefore, we intend to evaluate whether there is a significant difference in PFS between patients treated specifically with paclitaxel and paclitaxel+bevacizumab as a prespecified subgroup analysis.

### Sample size

The target number of patients was determined to compare PFS (the primary endpoint) between Groups A and B. Based on the findings of previous studies [5, 8, 25–33] in patients with recurrent ovarian cancer who became platinum resistant after the initial therapy or after the therapy for platinum-sensitive recurrent ovarian cancer, the median PFS of Group A (single-agent chemotherapy group) was assumed to be 3 months. Based on the initial-stage study plan of the AURELIA Study [8], the hazard ratio of Group B relative to Group A was assumed to be 0.7 (the median PFS of Group B corresponding to the hazard ratio of 0.7 is approximately 4.3 months, and the improvement rate in the median PFS is approximately 43%). These assumptions were made based on a clinically meaningful hazard ratio in platinum-resistant cases. The PFS of each group was assumed to follow an exponential distribution. When the allocation ratio to Groups A and B is set at 1:1, a total of 90 events will be required to ensure statistical power of ≥80% in the log-rank test with a one-sided significance level at 20%; this significance level was chosen because of the randomized phase II study design [34]. Assuming a registration period of 24 months, and a minimum PFS observation period of 6 months, 97 patients will be required in order to observe 90 events. The target number of patients in this study was determined to be 106 (approximately 53 per group), taking into account dropouts during the study period. East® Version 6.3 (Cytel, Inc., Cambridge, MA, USA) was used to calculate the sample size.

### Randomization

Patients will be allocated 1:1 by dynamic randomization (minimization method) to Groups A and B according to the following stratification factors: the number of regimens received in previous treatment (1, 2 vs. 3), the time to recurrence/disease progression from the last day of platinum-drug administration (during treatment vs. < 3 months vs. ≥3), and the chemotherapy drug (doxorubicin vs. topotecan vs. paclitaxel vs. gemcitabine). By including single-agent chemotherapy as an allocation adjustment factor for randomization, we believe that the type of chemotherapy will not affect the difference in outcome between the two groups.

### Statistical analysis

The primary analysis of PFS (primary endpoint) will be conducted at study completion or at the time point when at least 90 events of disease progression or death are observed, whichever occurs earlier. Secondary endpoints will be analyzed at the time of the primary analysis of the primary endpoint. The analysis populations will be the intent-to-treat (ITT) set, defined as patients who are registered to this study and randomly allocated to either of the groups; the per protocol set (PPS), defined as patients among the ITT set who have received the protocol therapy at least once and have no serious protocol deviation; and the safety analysis population, defined as patients who are registered to this study and received the protocol therapy at least once. The primary and secondary endpoints will be analyzed in the ITT set and PPS, and the analysis of the ITT set will be the primary analysis. Analysis of the safety endpoint will be conducted in the safety analysis population on an as-treated basis.

In each group, the median and quartile point of PFS and OS, and the 95% confidence intervals (two-sided) of the median PFS and OS will be estimated by the Kaplan–Meier method, and statistical significance between the groups will be evaluated by the stratified log-rank test with a significance level of 20% (one-sided) for PFS and 5% for OS. One-sided and two-sided *p*-values will be calculated to facilitate comparison of the study results with other studies. The probability of PFS at 3 and 6 months and that of OS at 6 and 12 months, and the corresponding 95% confidence intervals (two-sided), will be estimated. A Kaplan–Meier curve will be generated. A Cox proportional hazard model will be used for estimating the hazard ratio and its 95% confidence interval (two-sided). Groups alone will be the covariate of the Cox proportional hazard model, and the stratification factors previously described will be used for stratification. The Cox proportional hazard model will be used for a supplementary purpose to estimate the unadjusted hazard ratio and its 95% confidence interval (two-sided). Various sensitivity analyses will be conducted for the following three items to evaluate the robustness of the primary analysis: clinical progression not handled as a PFS event, cutoff at discontinuation of protocol therapy due to AEs or patient’s rejection, and cutoff on the day of starting post-therapy.

Regarding OS, patients without follow-up evaluation will be censored on the day of final dose of the protocol therapy. The patients without information on survival after baseline will be censored at the time of starting the protocol therapy.

ORR will be evaluated in patients in whom response is evaluable according to the RECIST v1.1 or GCIG CA125 criteria. A Cochran–Mantel–Haenszel (CMH) test will be used for evaluating the statistical significance between the groups, with a significance level of 5% (two-sided). ORR and its 95% Clopper–Pearson confidence interval (two-sided) will be estimated for each group. A logistic regression model will be used for estimating the adjusted odds ratio between treatment groups.

In each group, the number of patients who undergo paracentesis (number of patients with > 0 paracentesis procedures), the percentage of paracentesis procedures, and its 95% Clopper–Pearson confidence intervals (two-sided) will be estimated. Statistical significance between the groups will be calculated by the van Elteren test (stratified Wilcoxon’s rank sum test), with a significance level of 5% (two-sided).

The number and percentage of patients with AEs will be calculated in each group. The grades used for evaluation of AEs will be based on the CTCAE v4.0-JCOG [21]. AEs will be tabulated based on the Medical Dictionary for Regulatory Activities preferred terms by system organ class. The number of cycles, treatment period, and percentage of patients who received the scheduled dose for each drug and cycle will be summarized.

## Discussion

Available treatment options and the effectiveness of treatment in platinum-resistant recurrent ovarian cancer are very limited. Based on the GOG-0218 [6], OCEANS [7], and ICON7 [15] studies, there is a pressing need to establish treatment for patients who were previously treated with bevacizumab. The study described here is the first to assess the efficacy of bevacizumab beyond PD, particularly in platinum-resistant ovarian cancer patients previously treated with bevacizumab. The absence of quality of life assessment is a limitation of this study. However, because ours is the first study to address the clinical benefit of continued treatment with bevacizumab beyond progression in platinum-resistant recurrence, we believe it is more important to focus on safety and efficacy of such treatment. We intend to incorporate a quality of life endpoint into a subsequent phase III clinical study. The present trial and a phase III trial that will follow are of clinical significance in that these studies may show that combination therapy with bevacizumab and chemotherapy can be a new treatment option for ovarian cancer patients who were previously treated with bevacizumab.

## Abbreviations

AEs: Adverse events
CMH: Cochran–Mantel–Haenszel
ECOG: Eastern Cooperative Oncology Group
GCIG: Gynecological Cancer Intergroup
HBsAg: Hepatitis B surface antigen
ITT: Intent-to-treat
i.v.: Intravenously
JGOG: Japanese Gynecologic Oncology Group
ORR: objective response rate
OS: Overall survival
PD: Progressive disease
PFS: Progression-free survival
PPS: Per protocol set
RECIST: Response Evaluation Criteria In Solid Tumors
VEGF: Vascular endothelial growth factor
